# EANM/SNMMI guideline/procedure standard for [^18^F]FDG hybrid PET use in infection and inflammation in adults v2.0

**DOI:** 10.1007/s00259-024-06915-3

**Published:** 2024-10-10

**Authors:** Gad Abikhzer, Giorgio Treglia, Matthieu Pelletier-Galarneau, John Buscombe, Arturo Chiti, Elizabeth H. Dibble, Andor W. J. M. Glaudemans, Christopher J. Palestro, Mike Sathekge, Alberto Signore, Francois Jamar, Ora Israel, Olivier Gheysens

**Affiliations:** 1 Department of Medical Imaging, Faculty of Medicine and Health Sciences, Jewish General Hospital, McGill University, Montreal, Quebec Canada; 2 Nuclear Medicine, Imaging Institute of Southern Switzerland, Ente Ospedaliero Cantonale, Bellinzona, Switzerland; 3 Department of Nuclear Medicine and Molecular Imaging, Lausanne University Hospital, University of Lausanne, Lausanne, Switzerland; 4 Faculty of Biomedical Sciences, Università della Svizzera Italiana, 6900 Lugano, Switzerland; 5 Department of Medical Imaging, Montreal Heart Institute, Montreal, Quebec Canada; 6 Department of Nuclear Medicine, Cambridge University Hospitals, Cambridge, United Kingdom; 7 Department of Nuclear Medicine, IRCCS San Raffaele and Vita-Salute San Raffaele University, Milano, Italy; 8 Department of Diagnostic Imaging, Warren Alpert Medical School of Brown University, Rhode Island Hospital, Providence, RI USA; 9 Department of Nuclear Medicine and Molecular Imaging, University of Groningen, University Medical Center Groningen, Groningen, the Netherlands; 10 Department of Radiology, Zucker School of Medicine at Hofstra, Northwell, USA; 11 Nuclear Medicine Research Infrastructure (NuMeRI), University of Pretoria, Steve Biko Academic Hospital, Pretoria, South Africa; 12 Nuclear Medicine Unit, Department of Medical-Surgical Sciences and of Translational Medicine, University Hospital S. Andrea, “Sapienza” University, Roma, Italy; 13 Department of Nuclear Medicine, Cliniques Universitaires Saint-Luc and Institute of Clinical and Experimental Research (IREC), Université Catholique de Louvain, Brussels, Belgium; 14 Rappaport School of Medicine, Technion- Israel Institute of Technology, Haifa, Israel

**Keywords:** [^18^F]FDG, PET/CT, Inflammation, Infection, Guideline, Procedure standard

## Abstract

**Introduction:**

Hybrid [^18^F]FDG PET imaging is currently the method of choice for a wide variety of infectious and inflammatory disorders and was recently adopted in several clinical guidelines. A large amount of evidence-based articles, guidelines and appropriate use criteria have been published since the first version of this guideline in 2013.

**Purpose:**

To provide updated evidence-based information to assist physicians in recommending, performing and interpreting hybrid [^18^F]FDG PET examinations for infectious and inflammatory disorders in the adult population.

**Methods:**

A systematic literature search of evidence-based articles using whole-body [^18^F]FDG hybrid imaging on the indications covered within this guideline was performed. All systematic reviews and meta-analyses published within the last 10 years until January 2023 were identified in PubMed/Medline or Cochrane. For each indication covered in this manuscript, diagnostic performance was provided based on meta-analyses or systematic reviews. If not available, results from prospective or retrospective studies were considered based on predefined selection criteria.

**Results and conclusions:**

Hybrid [^18^F]FDG PET is extremely useful in the work-up and management of adults with infectious and inflammatory diseases, as supported by extensive and rapidly growing evidence-based literature and adoption in clinical guidelines. Practical recommendations are provided describing evidence-based indications as well as interpretation criteria and pitfalls. Monitoring treatment response is the most challenging but insufficiently studied potential application in infection and inflammation imaging.

**Supplementary Information:**

The online version contains supplementary material available at 10.1007/s00259-024-06915-3.

## Introduction

[^18^F]fluorodeoxyglucose ([^18^F]-2-fluoro-2-deoxyglucose or [^18^F]FDG) positron emission tomography with computed tomography (PET/CT) and magnetic resonance imaging (PET/MRI), further referred to as hybrid [^18^F]FDG PET imaging, are non-invasive diagnostic imaging procedures providing tomographic images for the determination of metabolic activity. The technology and radiotracers have been previously described in detail in the EANM guidelines for tumor imaging version 2.0 [1] and will be discussed only in the context of clinical indications discussed in the present document. Cells involved in infection and inflammation and their host response, especially neutrophils and the monocyte/macrophage family, result in increased [^18^F]FDG delivery to affected sites, up-regulation of glucose transporters, particularly GLUT1 and GLUT3, and increased hexokinase activity. Increased cell glycolysis occurs in both the acute and chronic inflammatory response [2]. The increased [^18^F]FDG uptake in infectious and inflammatory processes, its widespread availability, decreasing cost and ease of use, together with imaging devices that provide excellent sensitivity and resolution have led to the widespread use of [^18^F]FDG PET imaging for a variety of infectious and inflammatory diseases.

## Goals

The aim of this guideline is to provide general information about indications and protocols for hybrid [^18^F]FDG PET imaging in infection and inflammation in the adult population. Since the first version of this guideline was published in 2013 [3], there has been a rapidly growing use of [^18^F]FDG imaging in inflammation and infection, together with a large amount of published evidence-based articles, guidelines and appropriate use criteria on specific indications within this field. It has become evident that hybrid [^18^F]FDG metabolic imaging is nowadays the method of choice for most infection and inflammation indications. A systematic literature search of evidence-based articles using whole-body [^18^F]FDG imaging on the indications covered within this guideline was performed. All systematic reviews and meta-analyses on the topics listed in PubMed/Medline or Cochrane Library and published within the last 10 years until January 2023 were identified using the following search strings [(PET OR Positron OR FDG) AND (systematic review OR (meta-analysis)]. Results of reported systematic reviews and meta-analyses are based on publications including only PET/CT studies unless otherwise specified. Data from stand-alone PET devices were included only in indications when combined with PET/CT data in systemic reviews or meta-analyses in which separate data could not be extracted. An attempt to search for publications on the specific use of PET/MRI in this field resulted in only limited data, although it is expected that more data will be available in the future [4]. For each indication, we provide evidence for diagnostic performance based on systematic reviews or meta-analyses. When these are not available, results from prospective or retrospective studies are considered. As the use of [^18^F]FDG imaging in infection and inflammation is rapidly evolving, these guidelines cannot be seen as definitive and should be regarded as current advice. Therefore, the topics mentioned within this guideline also aim to identify further areas for clinical research when evidence is lacking at present.

This publication complements many EANM and SNMMI guidelines/procedure standards, which will be referenced in the appropriate sections, attempting to avoid duplication and replication of more specific recommendations on a particular topic such as information concerning PET/CT or PET/MRI performance and quality control, general acquisition parameters, radiopharmaceutical characteristics, and general basic and clinical aspects of [^18^F]FDG imaging addressed in topic-specific guidelines. The present guideline aims to provide physicians the knowledge and competence in the use of [^18^F]FDG imaging for infectious and inflammatory disorders. For each topic a short introduction will be followed by indications with and without sufficient evidence, diagnostic performance and areas of potential research. For certain indications, specific protocols and interpretation criteria will be covered with reference to existing procedural recommendations. Statements common to subsections for all or most of the topics will be covered in the second, general part of the document.

## Common clinical indications

### A. Fever and inflammation of unknown origin

Fever of unknown origin (FUO) is defined as fever higher than 38.3°C (100.9°F) persisting for at least 3 weeks, with no diagnosis despite 3 outpatient visits or in-patient days [5]. FUO is divided into four different subcategories: classical, nosocomial, neutropenic and Human Immunodeficiency Virus (HIV) – related. The etiology includes infectious, inflammatory, malignant and miscellaneous causes. The distribution varies according to the FUO subcategory and geographical location. Inflammation of unknown origin (IUO), defined as unexplained and prolonged elevation of inflammatory markers, without fever, shares similar etiologies [6]. [^18^F]FDG PET/CT has a high diagnostic yield in both these clinical settings. As many patients never have a final causative diagnosis, the diagnostic yield and helpfulness of [^18^F]FDG PET/CT are usually preferred over sensitivity and specificity.

#### Indications

- Evaluation of patients with FUO/IUO without a diagnosis despite standard work-up.

#### Indications with insufficient evidence

- Evaluation of patients with FUO and normal inflammatory markers (C-reactive protein-CRP, erythrocyte sedimentation rate-ESR).

#### Specific protocol points and interpretation criteria

- Consider myocardial suppression preparation (See section "Patient preparation and precautions, no.5") when there is a potential cardiac etiology.
- [^18^F]FDG PET/CT study should ideally be performed within 3 days of initiation of oral glucocorticoid therapy.

#### Diagnostic performance (Table 1)

Additional data:

- The results of [^18^F]FDG PET/CT can aid in identifying the etiology of FUO/IUO. It can guide further investigations, biopsy or specific treatment when the cause of the FUO/IUO is established.
- A negative [^18^F]FDG PET/CT can predict favorable prognosis through spontaneous remission of fever and potentially allows a watchful waiting approach [12].
- Cost effectiveness of [^18^F]FDG PET/CT, particularly when performed early in the diagnostic work-up, has been demonstrated in both FUO [13] and IUO [14].
  **Table 1:** Major performance values from systematic reviews and meta-analyses on [^18^F]FDG PET/CT in patients with FUO/IUO

Authors	No. studies (patients)	Sensitivity (95% CI)	Specificity (95% CI)	Comments
Hao et al. [7] (2013)	15 (595)	85% (81-88)	NR*	FUO
Takeuchi et al. [8] (2016)	22 (1137)	86% (81-90)	52% (36-67)	FUO
Bharucha et al. [9] (2017)	18 (905)	NR	NR	FUO Diagnostic yield: 56% (95% CI: 50-61)
Kan et al. [10] (2019)	23 (1927)	84% (79-89)	63% (49-75)	FUO & IUO Likelihood ratio +: 2.3 (1.5-3.4) and -: 0.25 (0.16 – 0.38) Diagnostic odds ratio (DOR): 9 (4-20)
Van Rijsewijk et al. [11] (2023)	54 (3192)	84% (NR)	62% (NR)	FUO & IUO Diagnostic accuracy 76%, helpfulness 61%

*NR – Not reported.

#### Areas of potential research

- Prospective studies on diagnostic yield, helpfulness and impact in patients with IUO.

### B. Infection

1. **Bacteremia/septicemia and evaluation of metastatic infection/septic embolism**

Bacteremia, the presence of viable bacteria in the bloodstream, can be incidental, occult, and non-life-threatening, but is often associated with severe illness. The term septicemia is used interchangeably with bacteremia but typically refers to a pathogenic organism in the bloodstream, frequently bacteria, which is associated with severe illness. Both entities are associated with high mortality and morbidity [15].

#### Indications

- Evaluation of the source of infection.
- Evaluation of septic emboli and metastatic infections.

#### Specific protocol points and interpretation criteria

- Myocardial suppression preparation should be performed.

#### Diagnostic performance (Table 2)

Additional data:

- A systematic review including 5 studies with 804 patients found low certainty of evidence that [^18^F]FDG PET/CT reduces mortality in patients with Staphylococcus aureus bacteremia [17].
- In a retrospective study of 30 intensive care patients with general bloodstream infections, [^18^F]FDG PET/CT had a sensitivity of 91%, specificity of 88%, positive predictive value (PPV) of 95%, and negative predictive value (NPV) of 78% [18].
- One study found [^18^F]FDG PET/CT to be cost effective in patients with gram positive bacteremia due to its ability to decrease mortality with a cost per prevented death within the range considered to be efficient by the authors’ national guidelines [19].
  **Table 2:** Major performance values of [^18^F]FDG PET/CT in a meta-analysis of critically ill patients with suspected infection

Authors	No. studies (patients)	Sensitivity (95% CI)	Specificity (95% CI)	Change in management (95% CI)	Contribution to diagnosis (95% CI)	DOR (95% CI)
Huang et al. [16] (2020)	4 (87)	94% (79-99)	66% (45-83)	41% (15-66)	65% (55-74)	2.8 (1.3-4.2)

#### Areas of potential research

- Prospective randomized trials on outcome in patients with bacteremia of unknown origin.

2.  **Suspected spondylodiscitis, with and without spinal hardware**

Spondylodiscitis, also referred to as spinal/vertebral osteomyelitis or spinal infection, involves the vertebral body (spondylitis) and disc (discitis). Clinical presentation typically includes fever and back pain. Bacteremia and endocarditis are among the most significant risk factors. In adults, the disc is avascular and is usually involved following an initial hematogenous septic embolus to the vertebral endplate. Infection can also extend to the posterior elements of the bone, pre- and para-vertebral soft tissues and the epidural space. Less commonly, spondylodiscitis can result from direct extension from adjacent soft tissue infection or direct inoculation during spinal procedures or penetrating trauma. A single level is involved in 65% of patients, while multilevel contiguous infection occurs in about 20% and non-contiguous infection in about 10% of patients [20]. A multi-society joint consensus document containing detailed evidence-based recommendations and a flow chart for the diagnosis of spondylodiscitis was published in 2019 [21]. Appropriate use criteria containing recommendations for selecting the imaging technique in musculoskeletal infections were published in 2021 [22].

#### Indications

- Suspected spondylodiscitis in patients without spinal hardware, particularly if MRI is contra-indicated.
- Suspected spondylodiscitis in patients with spinal hardware, preferably performed 3-4 months after surgery.
- Suspected spondylodiscitis with inconclusive/indeterminate MRI and elevated inflammatory markers (ESR and/or CRP).
- Evaluation of multi-level spondylodiscitis.
- Identification of the source and/or extent of dissemination of infection in established spondylodiscitis.

#### Specific protocol points and interpretation criteria

- Sagittal views should routinely be assessed to analyze the spine.
- In patients without hardware, spondylodiscitis appears as:
  o Focal or linear increased uptake in one or adjacent vertebral endplates above the intensity of normal bone marrow activity.
  o Increased uptake in the adjacent disc space and/or paravertebral soft tissues may also be present.
- Extension of abnormal uptake, particularly to the epidural space, should be assessed.
- Correlative CT findings supportive for infection: end-plate irregularities, erosions and/or destruction, extension to adjacent soft tissues or presence of collections. However, these findings may be absent early in the disease.
- In patients with spinal hardware:
  - Spondylodiscitis appears as intense, confluent uptake in the soft tissues and bone adjacent to the hardware at multiple contiguous levels, potentially extending to the bone-hardware interface of one or more inter-pedicular screws.
  - Aseptic inflammation in loosening and bone remodeling appears as uptake adjacent to one or two hooks, screws or anchors, usually at the upper or lower portions of the hardware.

#### Diagnostic performance (Table 3)

Additional data:

- [^18^F]FDG PET/CT could be the modality of choice for detection of spondylodiscitis in patients within 14 days of symptom onset [24].
- According to a retrospective study, [^18^F]FDG PET/CT changed management in 52% of patients with spondylodiscitis, including starting or modifying antibiotic therapy, or guiding biopsy and surgical interventions [25].
  **Table 3:** Major performance values in a single systematic review on [^18^F]FDG PET/CT in patients with suspected spinal infections

Authors	No. of studies (patients)	Sensitivity (95% CI)	Specificity (95% CI)	Likelihood ratio + (95% CI)	Likelihood ratio – (95% CI)	DOR (95% CI)
Treglia et al. [23] (2020)	26 (833)	95% (89-98)	91% (78-97)	4.7 (2.9-7.7)	0.11 (0.07-0.16)	63.4 (28.9-139)

#### Areas of potential research

- Understanding the kinetics of post-operative [^18^F]FDG uptake following spinal surgery with metallic hardware to improve specificity of interpretation.
- Prospective studies to assess the added value of PET/MRI for the diagnosis of spondylodiscitis.

3.  **Suspected non-complicated osteomyelitis and septic arthritis (excluding diabetic foot and spine)**

Osteomyelitis is caused by direct extension from trauma and/or surgery or by hematogenous spread from a remote source [22]. In the acute phase, infection can usually be recognized clinically. In the chronic phase, signs and symptoms are often non-specific. Imaging procedures performed routinely as part of the diagnostic workup consist of radiographs followed by MRI, labelled white blood cells (WBC) scintigraphy with SPECT/CT or [^18^F]FDG PET/CT [26].

Septic arthritis is the involvement of a single or multiple joints and synovial fluid by an infectious pathogen. Risk factors are among others, diabetes mellitus and HIV. It is more common in patients with rheumatoid arthritis or a prosthetic joint [22]. Early diagnosis is essential to initiate prompt and adequate treatment to avoid destruction of cartilage.

#### Indications

- Suspicion of bone involvement in cases of a known soft tissue infection.
- In specific cases, to assess for dissemination of infection to other skeletal sites or organs.

#### Indications with insufficient evidence

- Differentiation between infectious and non-infectious/inflammatory arthritis.

#### Specific protocol points and interpretation criteria

- Infection appears as heterogeneous, intense, focal uptake in the bone, usually adjacent to and extending from a soft tissue infection in cases of osteomyelitis or in the joints, in cases of septic arthritis.
- Absence of joint [^18^F]FDG uptake can exclude septic arthritis.
- Adjacent bones should be assessed for involvement in selected cases with suspected dissemination of infection.

#### Diagnostic performance (Table 4)

No systematic reviews specifically addressed the role of [^18^F]FDG PET/CT for the diagnosis of uncomplicated osteomyelitis or septic arthritis.

**Table 4:** Major performance values of single systematic review of [^18^F]FDG PET and PET/CT for diagnosis of osteomyelitis

Authors	No. studies (patients)	Sensitivity (95% CI)	Specificity (95% CI)	DOR (95% CI)
Llewellyn et al. [27] (2019)	16 (656)	85% (72-93)	93% (83-97)	38.5 (17.8-83.3)

#### Areas of potential research

- Assessing whether [^18^F]FDG PET/CT can be used as the standard diagnostic modality for uncomplicated peripheral bone osteomyelitis or whether it requires confirmation with other tests.

4.  **Suspected osteomyelitis (excluding diabetic foot, prosthesis and spine) in complicated bone**

Osteomyelitis can occur in bones that were previously violated by fractures, surgery, and/or metallic hardware. Diagnosis of infection in this setting is difficult. Persistent pain can be multifactorial, due to healing, inflammation and/or infection. Bone may be regenerating, fracture healing may be hampered, and the bone structure may be inadequate, affecting the image quality and interpretation. In these cases, functional nuclear medicine tests are better suited than radiological modalities for the diagnosis of complicated osteomyelitis [28].

#### Indications

- Suspected (1) fracture-related infection, with and without metallic hardware and (2) sternal wound infection, including mediastinitis:
  o To differentiate between osteomyelitis and reactive inflammation.
  o To define the extent of infection.
  o To guide appropriate treatment strategies such as the need for and planning of surgery.
  o To assess for dissemination of infection to other skeletal sites or organs in selected cases.

#### Specific protocol points and interpretation criteria

- Acquisition FOV:
  - In suspected sternal wound infection and/or mediastinitis, the whole-body study should ideally be acquired with arms above the head.
- Patterns of infection [29]:
  - Osteomyelitis: heterogenous/focal uptake localizing to bone.
  - Fracture-related infection: heterogenous/focal uptake at the fracture site, extending to adjacent soft tissue or focally involving metallic hardware interface when present.
    - Non-infectious patterns: diffuse homogenous uptake confined to the fracture line. Intensity of uptake can vary with time since the occurrence of the fracture or healing complications. Uncomplicated fracture uptake usually normalizes within 3 months of trauma [30].
    - Homogenous mild uptake along the surface or tip of a metallic implant can persist in non-infected hardware.
  - Sternal osteomyelitis: sternal focal uptake, extending to adjacent soft tissue or sternal wire uptake [31].
    - Diffuse, homogeneous uptake confined to the sternum can persist for months to years after surgery without any signs of infection.

Diagnostic performance

#### Fracture-related infection (Table 5)

Additional data:

- [^18^F]FDG PET/CT performed within the first month after surgery was found to be an independent variable with the highest predictive value for a false positive test result [33].
- No systematic review specifically addressed the role of [^18^F]FDG PET/CT for diagnosing metallic hardware infection. In a single retrospective study including more than 20 patients with metallic hardware, sensitivity was 88%, specificity 76% and diagnostic accuracy 82% [34].
  **Table 5:** Major performance values of systematic reviews or meta-analyses on [^18^F]FDG PET/CT in patients with fracture-related infections

Authors	No. studies (patients)	Sensitivity (95%CI)	Specificity (95% CI)	Area Under the Curve (95% CI)	Likelihood ratio + (95% CI)	Likelihood ratio - (95% CI)	DOR (95% CI)
Govaert et al. [28] (2017)	3 (NR)	86-94%	76-100%	NR	NR	NR	NR
Zhang et al. [32] (2021)	6 (NR)	89% (81-94)	78% (72-84)	0.93 (0.90-0.95)	4.1 (3.1-5.4)	0.14 (0.08-0.25)	29 (14-61)

#### Sternal infection, including mediastinitis

- No systematic review specifically addressed the role of [^18^F]FDG PET/CT for diagnosing bone infection and/or mediastinitis after sternal surgery.
- Available studies [31, 35] reported diagnostic performance indices of [^18^F]FDG PET/CT for:
  - Sternal osteomyelitis: sensitivity 91-98%, specificity 95-97%.
  - Mediastinitis: sensitivity 78%, specificity 82%.

#### Areas of potential research

- Standardized interpretation criteria for diagnosis of complicated osteomyelitis based on pattern and intensity of uptake.
- Determine [^18^F]FDG PET/CT criteria for the evaluation of response to conservative antibiotic treatment and to surgery, including debridement.

5. **Diabetic foot infections**

Diabetic patients are predisposed to severe foot infections, including osteomyelitis in addition to soft tissue involvement, which are associated with high morbidity and increased mortality. Evidence-based guidance for imaging of the diabetic foot have recently been published [36, 37].

#### Indications

- Diagnosis of diabetic foot osteomyelitis in patients with a soft tissue ulcer and high suspicion of bone involvement after clinical examination, probe to bone test and radiographs [22].

#### Indications with insufficient evidence

- Differentiation of osteomyelitis in diabetic patients with mid- and hind-foot ulcers from superimposed active diabetic neuropathic osteoarthropathy (Charcot) [38].

#### Specific protocol points and interpretation criteria

- Acquisition FOV can be limited to the feet, except in cases of sepsis/bacteremia.
- Use of a foot holder to prevent movement is recommended.
- Additional parameters are specified in sections "Image acquisition protocol" and "Image analysis and interpretation".
- Soft tissue infection: focal or diffuse uptake localized only to soft tissues without extension to bone.
- Osteomyelitis: focal or diffuse uptake, regardless of SUV, localized to bone, extending contiguously and/or tracking from an adjacent infected soft tissue ulcer.
- Diabetic foot neuropathic-osteoarthropathy (Charcot): diffuse uptake localized to joints, usually in the mid- and/or hind-foot.
- CT: bones adjacent to the soft tissue infection should be assessed for erosions or osseous destruction.

#### Diagnostic performance (Table 6)

**Table 6:** Major performance values of meta-analyses for diabetic foot osteomyelitis with [^18^F]FDG PET or PET/CT

Authors	No. studies (patients)	Sensitivity (95% CI)	Specificity (95% CI)	Likelihood Ratio +(95% CI)	Likelihood Ratio -(95% CI)	DOR (95% CI)
Treglia et al. [39] (2013)	4 (178)	74% (60-85)	91% (85-96)	5.6 (2-15.3)	0.37 (0.1-1.35)	16.9 (2-139.6)
Lauri et al. [40] (2017)	6 (254)	89% (68-97)	92% (85-96)	11 (4.7-25)	0.11 (0.03-0.4)	95 (18-504)
Llewellyn et al. [41] (2020)	6 (NR)	84% (53-96)	93% (76-98)	NR	NR	33.9 (12-98)

#### Areas for potential research

- Validation of standardized interpretation criteria in a multi-center study.
- Define the role of [^18^F]FDG PET/MRI for the diagnosis of diabetic foot osteomyelitis, as well as for differentiation from diabetic neuropathic osteoarthropathy.

6. **Peri-prosthetic joint infections**

Peri-prosthetic joint infections (PJI) involve prostheses and adjacent soft tissues. Patients usually present with joint pain. Fever and erythema are less common. Laboratory tests show elevated inflammatory markers, CRP and/or ESR. Diagnosis of PJI is based on a combination of blood, synovial and tissue sample analyses. When the diagnosis is clinically challenging, functional imaging can provide useful information [42].

Appropriate use criteria for musculoskeletal infections include a detailed analysis of optimal imaging test selection [22]. A multi-society joint consensus document published in 2019 contains detailed evidence-based recommendations and a flow chart with respect to the diagnosis [43], role and choice of imaging tests in PJI [44].

#### Indications

- Suspected PJI of the hip in patients in whom an imaging test with high sensitivity is clinically necessary (preferably at least 2 years after surgery [43]).
- To rule out PJI in selected cases with a low pre-test probability of infection, e.g. before revision surgery.

#### Indications with insufficient evidence

- Utility for diagnosis of PJI at other sites, such as the knees [45, 46], shoulders [43] and ankles.

#### Specific protocol points and interpretation criteria

- See sections "Image acquisition protocol" and "Image analysis and interpretation".
- Acquisition FOVs can usually be limited to the prosthetic region, except in cases of sepsis/bacteremia.
- The date of surgery and type of prosthesis should be considered when interpreting studies.
- Absence of peri-prosthetic uptake reliably excludes infection in both hip and knee prostheses [44].
- The presence of peri-prosthetic uptake should be interpreted with caution.
  - High specificity may be difficult to obtain. The differential diagnosis of increased uptake can include recent surgery, peri-prosthetic inflammation, foreign body reaction, aseptic loosening, fractures, metal-related disease, pseudo-tumors and malignancy.
  - WBC scintigraphy may be obtained when [^18^F]FDG PET/CT results are inconclusive.

*PJI of the hip:*

- Various interpretation criteria for PJI have been proposed with variable results [47–51].
  o Uptake at the bone-prosthesis interface, particularly when associated with peri-prosthetic soft tissue uptake.
  o Extensive, heterogeneous uptake in collections or intramuscular fluid.
  o The presence of a sinus tract is infection specific.
- Non-infected hip prostheses can show heterogeneous uptake at and adjacent to the femoral head and/or neck portion of the prosthesis, particularly in the greater trochanteric region [52–54].
- CT: presence of soft tissue collections, fluid filled bursae, joint distension, fluid collections in muscles and adjacent fat, support, but are not diagnostic of infection. Absence of joint distension has a high NPV for infection. New periosteal bone reaction is a specific finding. Bone lucency surrounding the prosthesis is non-specific unless severe [44].

#### Diagnostic performance (Table 7)

Additional data:

- In a study comparing the various criteria for PJI of the hip, uptake along the femoral bone-prosthesis interface (Zone B-mid femur) showed the highest diagnostic performance with a sensitivity of 81% and specificity of 84% [51].
  **Table 7:** Major performance values of systematic reviews and meta-analyses on [^18^F]FDG PET or PET/CT in patients with suspected peri-prosthetic joint infections

Authors	No. studies (prostheses)	Sensitivity (95%CI)	Specificity (95%CI)	Area Under Curve	Likelihood Ratio + (95%CI)	Likelihood Ratio - (95%CI)	DOR (95%CI)
Jin et al. [55] (2014)	14 (838)	86% (82-90)	86% (83-89)	0.93	NR	NR	NR
Verberne et al. [56] (2016)	12 (725)	69% (58-79)	96% (93-98)	NR	NR	NR	NR
Verberne et al. [45] (2017)	5 (179)	70% (56-81)	84% (76-90)	NR	NR	NR	NR
Kim et al. [57] (2021)	19 (826)	88% (80-93)	89% (83-93)	0.94	7.9 (5.1-12.2)	0.14 (0.08-0.23)	57 (31-106)
Hu et al. [46] (2022)	23 (1437)	85% (76-91)	86% (78-91)	0.92	6.1 (3.8-9.7)	0.17 (0.11-0.28)	35 (17-74)

#### Areas of potential research

- Multicenter studies to define and validate the optimal imaging time post prosthesis implantation to reduce false positive [^18^F]FDG PET/CT results, as well as the uptake variability dependant on type of prosthesis used (e.g. cemented vs. non-cemented).
- Validation and reproducibility of standardized interpretation criteria for diagnosis of PJI for different sites, including assessing the variability and potential patterns of uptake in painful, non-infected prostheses in different joints, such as shoulders, hips, knees, ankles.

7. **Prosthetic valve endocarditis**

Prosthetic valve endocarditis (PVE) represents 20% of all cases of infective endocarditis (IE) and is associated with high morbidity and mortality [58, 59]. Accurate, early diagnosis is critical to guide management, which frequently involves removal of the infected material and implantation of a new valve. The yield of the modified Duke criteria for PVE is limited by the low sensitivity of echocardiography and frequent negative blood cultures as compared to native valve endocarditis (NVE). [^18^F]FDG PET/CT has been included as a major criterion for the diagnosis of IE in clinical practice guidelines [60, 61].

#### Indications

- Suspected PVE with negative or inconclusive echocardiography [60, 62].
- Detection of septic emboli/metastatic infections when PVE is suspected or established [60, 62].
- Detection of the primary source of infection when the diagnosis of PVE is established [62].
- Therapy response assessment in PVE patients on conservative medical treatment due to contraindication to surgery [60].

#### Specific protocol points and interpretation criteria

- Myocardial suppression protocol is critical for patient preparation.
- Acquisition FOV encompassing the vertex-to-toes is recommended to evaluate for suspected emboli and to identify the source of bacteremia.
- Performing CT angiography (CTA) as part of the study can reduce the number of equivocal cases.
- Image reorientation in the valve plane allows for better assessment of [^18^F]FDG distribution.
- PVE: focal or heterogeneous uptake on or adjacent to the prosthetic valve [60].
- Non-infected prosthetic valves: homogeneous diffuse uptake of the valve, which can persist indefinitely after surgery.
  o Use of surgical adhesives may result in focal uptake and should be considered during interpretation [63, 64].
- In selected cases of suspected PVE with indeterminate [^18^F]FDG PET/CT uptake, a positive WBC SPECT/CT could confirm PVE, although a negative study cannot rule out the diagnosis.

#### Diagnostic performance (Table 8)

Additional data:

- In a multicenter prospective study including 175 patients, adding [^18^F]FDG PET/CT as a major criterion to the Duke criteria increased the sensitivity from 57% to 84% and decreased specificity from 96% to 71% mainly by reclassifying ‘Possible IE’ to ‘Definite IE’ [68].
- Lower CRP levels are associated with higher rates of false negative studies [63, 69].
- A single center prospective study, combining [^18^F]FDG PET with CTA had superior diagnostic performance compared to [^18^F]FDG PET/CT, mainly by reducing the proportion of equivocal cases from 20% to 8% [70].
- In a prospective multicenter study, including both NVE and PVE, 35% of patients had extra-cardiac findings on [^18^F]FDG PET/CT, such as spondylodiscitis or malignancy, leading to a change in management in 10% of cases [71].
- In the prospective multicenter TEPvENDO study, modification of management occurred in 21% of patients following [^18^F]FDG PET/CT, mainly due to documentation of perivalvular uptake [72].
- In a prospective study of 109 patients with 1-year follow-up, [^18^F]FDG PET/CT predicted adverse events defined as a composite of death, recurrence, acute cardiac failure, hospitalization, and new embolic events [69].
- In a single center prospective cohort study, systematic utilization of [^18^F]FDG PET/CT was associated with a 2-fold reduction of relapse [73].
  **Table 8:** Major performance values of meta-analyses for PVE with [^18^F]FDG PET/CT

Authors	No. studies (patients)	Sensitivity (95% CI)	Specificity (95% CI)	Likelihood Ratio + (95% CI)	Likelihood Ratio - (95% CI)	DOR (95% CI)
Juneau et al. [65] (2018)	7 (NR)	85% (77-91)	81% (72-88)	NR	NR	NR
Mahmood et al. [66] (2019)	8 (NR)	81% (74-86)	73% (64-81)	NR	NR	NR
Wang et al. [67] (2020)	15 (634)	86% (81-89)	84% (79-88)	3.23 (1.75-5.95)	0.21 (0.14-0.32)	22 (10-48)

#### Areas of potential research

- Added value of delayed static cardiac images, gated acquisition. and the addition of CTA in multicenter studies.

8.  **Native valve endocarditis**

NVE affects approximately 10-15/100,000 persons per year [74]. Its incidence increases in the presence of risk factors such as valvular abnormalities (e.g. bicuspid aortic valve), previous history of NVE and intravenous drug use. Diagnosis of NVE is based on the modified Duke Criteria, which include major and minor criteria composed of clinical and para-clinical findings such as blood cultures and echocardiography findings [60]. Abnormal [^18^F]FDG native valvular uptake was included as a major criterion in the 2023 update of the Duke’s criteria. Septic emboli can be detected in 15-45% of patients with suspected IE [67, 75].

#### Indications

- FUO or bacteremia with suspected NVE.
- Detection of septic emboli/metastatic infections in suspected or confirmed NVE.
- Detection of source of infection in suspected or confirmed NVE [60].

#### Indications with insufficient evidence

- Diagnosis of NVE when echocardiography is inconclusive or negative but with persistent high clinical suspicion.

#### Specific protocol points and interpretation criteria

- Myocardial suppression protocol is critical for patient preparation.
- Acquisition FOV encompassing the vertex-to-toes is recommended to evaluate for suspected emboli and to identify the source of bacteremia.
- Static delayed imaging of the heart can be performed at more than 90 minutes post injection, especially in equivocal cases [75].
- NVE: focal uptake projecting in the valve plane, regardless of intensity.
  o Focal papillary muscle uptake, and incomplete myocardial suppression patterns should be distinguished from valvular uptake.
- Valvular calcifications can demonstrate mild diffuse uptake.
- Septic emboli represent an indirect sign of NVE and are considered minor criteria.
- Diffuse increased splenic uptake may represent an indirect sign of active infection and increases the likelihood of NVE [60, 76].

#### Diagnostic performance (Table 9)

Additional data:

- Compared to PVE, the sensitivity of [^18^F]FDG PET/CT for NVE is historically very low, but the specificity is very high [65–67].
- Utilization of appropriate study preparation and contemporary PET/CT devices increases sensitivity [77].
- In the prospective multicenter TEPvENDO study, modification of management occurred in 31.4% of patients with NVE following [^18^F]FDG PET/CT, mostly due to extra-cardiac findings^72^.
- In a prospective study including 64 NVE patients with a 1-year follow-up, [^18^F]FDG PET/CT was an independent predictor of new embolic events [69].
- [^18^F]FDG PET/CT can provide additional data when surgical intervention is considered in patients with large vegetations [69].
  **Table 9:** Major performance values of meta-analyses for NVE with [^18^F]FDG PET/CT

Authors	No. studies (patients)	Sensitivity (95% CI)	Specificity (95% CI)	Likelihood Ratio + (95% CI)	Likelihood Ratio - (95% CI)	DOR (95% CI)
Wang et al. [67] (2020)	4 (297)	31% (21-41)	98% (95-99)	14.0 (5.6-35.4)	0.71 (0.60-0.84)	23.0 (8.1-65.6)
Kamani et al. [77] (2020)	7 (351)	36% (22-54)	99% (89-100)	8.3 (3.7-18.3)	0.6 (0.27-1.33)	15.3 (6.1-38.4)

#### Areas of potential research

- To assess if sensitivity can be improved with digital PET/CT, delayed static or gated cardiac studies and PET/CTA.

9. **Cardiac implantable electronic device infection**

Cardiac implantable electronic devices (CIEDs) include pacemakers, implantable cardiac defibrillators (ICD), and cardiac resynchronization therapy (CRT) devices. While relatively rare, with an incidence ranging between 0.6 and 3.4%, CIED infections are associated with significant morbidity, mortality, and healthcare costs [78]. Prompt removal of infected devices is associated with shorter hospitalization durations and lower in-hospital mortality, hence the importance of early diagnosis [79]. Reporting on lead/valve involvement is important as it may change the therapeutic approach.

#### Indications

- Suspected CIED infection [78].
- FUO or sustained bacteremia in patients with CIED to identify a potential source of infection.
- Diagnosis of deep CIED pocket infection.
- Suspected CIED related endocarditis [60, 62].
- Assessment of disease dissemination in patients with CIED infection.

#### Specific protocol points and interpretation criteria

- Myocardial suppression preparation is critical to accurately assess intra-cardiac leads and valves.
- Delayed acquisition may improve sensitivity to detect lead infection without compromising specificity [80].
- Deep pocket infection: intense, focal or heterogeneous uptake posterior to the generator.
- Lead infection: focal uptake along the leads.
- Increased [^18^F]FDG uptake in the immediate post implantation period surrounding the pocket and the proximal lead is to be expected.

#### Diagnostic performance (Table 10)

Additional data:

- In a prospective study including 105 patients with CIED infection, [^18^F]FDG imaging increased the sensitivity to detect CIED-related IE and predicted long-term survival [83].
- The sensitivity to detect CIED related infection is higher when using appropriate preparation protocols [81, 82].
  **Table 10:** Summary of major performance values of meta-analyses for CIED infections with [^18^F]FDG PET/CT

Authors	No. studies (patients)	Sensitivity (95% CI)	Specificity (95% CI)	Likelihood Ratio + (95% CI)	Likelihood Ratio – (95% CI)	DOR (95% CI)
Cardiac Implantable Electronic Device Infection Overall						
Juneau et al. [81] (2017)	11 (340)	87% (82-91)	94% (88-98)	NR	NR	NR
Mahmood et al. [82] (2019)	14 (492)	85% (80-89)	90% (84-94)	NR	NR	NR
Cardiac Implantable Electronic Device Infective Endocarditis /Lead Infection						
Juneau et al. [81] (2017)	6 (133)	65% (53-76)	88% (77-94)	NR	NR	NR
Mahmood et al. [82] (2019)	7 (NR)	76% (65-85)	83% (72-90)	NR	NR	NR
Wang et al. [67] (2020)	9 (208)	72% (61-81)	83% (75-89)	5.3 (1.4-19.4)	0.36 (0.19-0.69)	18 (4.7-68.9)
Cardiac Implantable Electronic Device Generator/Pocket Infection						
Juneau et al. [81] (2017)	4 (115)	93% (84-98)	98% (88-100)	NR	NR	NR
Mahmood et al. [82] (2019)	3 (NR)	96% (86-99)	97% (86-99)	NR	NR	NR

#### Areas of potential research

- Define the diagnostic and prognostic performance for different device subtypes (e.g. subcutaneous defibrillator).

10. **Ventricular Assist Device Infection**

Ventricular assist devices (VAD) are utilized for the management of end-stage heart failure as a bridge to transplantation, for destination therapy in patients not eligible for transplantation, and as a bridge to recovery [84]. VAD infection is relatively frequent and can affect any component of the device, with an incidence of 37/100 VAD patient-years [85]. Management strategy is guided by the severity of infection and the components involved. Superficial infection can be treated conservatively while deep infection may require surgical debridement or device explantation [86].

#### Indications

- Evaluation of driveline, pump, or cannula infection.
- FUO or bacteremia in VAD patients.
- VAD patients with embolic events of unknown source.
- Assessment of infection source and disease dissemination.

#### Specific protocol points and interpretation criteria

- Myocardial suppression preparation protocol is necessary.
- Acquisition encompassing the head and lower limbs is recommended to evaluate for dissemination and identification of the source of bacteremia.
- VAD infection: focal or heterogeneous uptake.
- Uninfected LVAD: may show homogeneous uptake, more intense at the left ventricular apex.
- Use of adhesives/biological glue may lead to focal uptake, especially at the inflow and outflow cannula.

#### Diagnostic performance (Table 11)

Additional data:

- [^18^F]FDG PET/CT can detect and localize VAD-related infection and predict clinical outcomes based on the location of infection [89]:
  o Driveline infection: sensitivity 97%, specificity 99% [88].
  o Pump/pocket infection: sensitivity 97%, specificity 93% [88].
- In a prospective study including 57 LVAD recipients, a positive [^18^F]FDG PET/CT study was associated with adverse outcome, including mortality. Involvement of the pocket was associated with worse outcomes [90].
- In a small retrospective study, [^18^F]FDG PET/CT altered medical management in 12 out of 21 patients [91].
  **Table 11:** Major performance values of meta-analyses for VAD infections with [^18^F]FDG PET/CT

Authors	No. studies (patients)	Sensitivity (95% CI)	Specificity (95% CI)	Likelihood Ratio + (95% CI)	Likelihood Ratio – (95% CI)	DOR (95% CI)
Tam et al. [87] (2020)	4 (95)	92% (82-97)	83% (24-99)	NR	NR	NR
Ten Hove et al. [88] (2021)	8 (256)	95% (89-97)	91% (54-99)	3.5 (1.8-6.9)	0.14 (0.10-0.18)	38.4 (NR)

#### Areas of potential research

- Diagnostic accuracy for latest generation VADs.
- Diagnostic accuracy for fungal VAD infection.

11. **Vascular graft and endograft infections (VGEI)**

Diagnosis of vascular graft and endograft infections (VGEI) is usually made with the help of clinical and imaging findings, and microbiological examinations. The clinical presentation varies from mild to severe symptoms. The Management of Aortic Graft Infection (MAGIC) group has developed a list of major and minor criteria with respect to clinical, surgical, radiological, and laboratory findings [92]. CTA is the first-line imaging method. A second-line imaging test such as [^18^F]FDG PET/CT or WBC scintigraphy is useful in cases with equivocal or even negative CTA and a high clinical probability [92, 93]. Recently published clinical [92] and EANM imaging [93] guidelines on VGEI provide detailed recommendations regarding the use of [^18^F]FDG PET/CT.

#### Indications

- Diagnosis of VGEI in the presence of at least one major clinical or laboratory MAGIC criterion with negative or doubtful CTA results and persisting clinical suspicion (preferably at least 4 months after surgery) [93].
- Diagnosis of suspected VGEI in the presence of at least two minor clinical or laboratory MAGIC criteria (lower pre-test probability) independently from the results of a previous CTA [93].
- Evaluation of the extent of VGEI.

#### Specific protocol points and interpretation criteria

- Suggested visual scoring interpretation criteria, none being universally accepted [93].
  o Five-point visual scale: 1: normal background activity; 2: mild, diffuse uptake along graft; 3: focal/mild or intense/diffuse uptake along graft; 4: focal and intense uptake; 5: focal and intense uptake associated with fluid collections. Score values of 3-5 are positive for VGEI [94].
  o Six-point visual scale: 1: normal background activity; 2: homogeneous, diffuse uptake of any intensity along graft; 3: non-homogeneous, diffuse uptake of any intensity not uniformly distributed along the graft; 4: focal uptake of any intensity; 5: focal and diffuse uptake of any intensity with ≥ 1 focal area clearly detectable; 6: uptake extending to peri-prosthetic tissues. Score values of 4-6 are positive for VGEI [95].
- Pitfalls:
  o Physiological uptake or activity in sterile inflammation/foreign body reaction that may persist indefinitely and depends on the prosthetic material used: diffuse, homogeneous uptake along the graft [93].
  o False positive findings can occur within the first 4 months after surgery, due to a difficult differential diagnosis between physiological sterile/postoperative inflammation and an infected or thrombosed graft. In the later post-surgical phase, uptake decreases when there is less sterile inflammation.

#### Diagnostic performance (Table 12)

Additional data:

- [^18^F]FDG PET/CT can detect unknown incidental findings with impact on management in about 40% of patients with suspected VGEI [101].
-
  **Table 12:** Major performance values from meta-analyses on diagnostic accuracy of [^18^F]FDG PET/CT in suspected VGEI

Authors	No. studies (patients)	Sensitivity (95% CI)	Specificity (95% CI)	Likelihood Ratio + (95% CI)	Likelihood Ratio – (95% CI)	DOR (95% CI)	Comments (PET interpretation criteria)
Reinders Folmer et al. [96] (2018)	5 (144)	95% (87-99)	80% (69-89)	NR	NR	38.0 (8.5-170.4)	NR
Kim et al. [97] (2019)	10 (286)	96% (89-98)	74% (67-81)	3.7 (2.9-4.9)	0.06 (0.02-0.15)	63 (23-173)	
Rojoa et al. [98] (2019)	8 (NR)	97% (89-99)	89% (70-96)	NR	NR	NR	Focal uptake
		97% (77-99)	62% (31-86)	NR	NR	NR	Graded uptake
		99% (95-99)	78% (68-86)	NR	NR	NR	SUVmax
Reinders Folmer et al. [99] (2020)	13 (415)	90% (79-96)	59% (38-78)	NR	NR	10.7 (3.4-33.6)	Uptake intensity
		94% (89-97)	81% (71-88)	NR	NR	52.4 (19.4-141.6)	Uptake pattern
		95% (76-99)	77% (63-87)	NR	NR	30.9 (7.3-130.8)	SUVmax
Mahmoodi et al. [100] (2022)	10 (320)	92% (88-95)	76% (70-81)	3.5 (2.3-5.3)	0.14 (0.09-0.23)	37.1 (14.8-92.8)

12.  **Suspected infected liver and kidney cysts**

Liver and renal cysts can become infected primarily by gram negative bacteria [102]. Patients with autosomal dominant polycystic kidney disease (ADPKD) who have suffered renal failure and required transplantation are at higher risk for liver and kidney cyst infection. Use of [^18^F]FDG PET/CT has been reported in only 3 papers with more than 20 patients, all with underlying ADPKD. None of these papers reported infection of liver or kidney cysts separately [103–105].

#### Indications

- To identify infected liver and/or renal cysts, primarily in patients with ADPKD.

#### Indications with insufficient evidence

- Suspected infected echinococcus liver cysts [106].

#### Specific protocol points and interpretation criteria

- If registration is poor and cannot be corrected, consider rescanning the FOV containing the liver and/or kidneys (as appropriate) with the CT performed in shallow breathing or mid-breath hold or using intrinsic or extrinsic respiratory gating acquisitions.
- Any focal [^18^F]FDG uptake with an intensity above liver uptake should be localized using the CT to identify the structure involved. An uptake equal or higher than liver background is considered positive for infection.

#### Diagnostic performance

- The evidence is based on single center retrospective studies [103–105]. In a summed total of 122 patients with suspected liver or kidney cysts infection, the overall sensitivity and PPV was 79% and the specificity and NPV 78%. There was not enough available information to calculate performance indices for liver and kidney cyst infections separately.
  o If only uptake in the cyst wall higher than liver background is considered as positive for infection, the specificity increased to 85% [103].

#### Areas of potential research

- To define optimal protocols for imaging infected liver and renal cysts in order to improve sensitivity.
  o For example, in patients with residual renal function, whether administration of diuretics can differentiate physiological accumulation in communicating renal cysts from an infected cyst.
  o In patients with suspected infected liver echinococcus cysts, evaluate if delayed acquisition can improve sensitivity.

13. **Invasive fungal infections**

Invasive fungal infections (IFI) occur mainly in immunosuppressed patients. A role of [^18^F]FDG PET/CT in this setting has been suggested. The intensity of uptake helps stage IFI, and the resolution of the uptake as result of the healing process forms the basis of monitoring therapy. To monitor antifungal treatment with [ [18]F]FDG PET/CT, at least two studies performed at different times while the patient is on antifungal treatment are required [107].

#### Indications with insufficient evidence

- Assessment of disease extent and monitoring response to therapy.

#### Diagnostic performance

- There are no systematic reviews assessing the role of [^18^F]FDG PET/CT in patients with invasive fungal infections at diagnosis and following therapy.
- One prospective study reported the presence of increased uptake in sites of IFI identified by conventional imaging. In addition, [^18^F]FDG PET/CT detected small IFI lesions not seen on conventional imaging. Uptake disappeared after 6 months of antifungal therapy in some of the patients [108].
- A prospective study concluded that baseline [^18^F]FDG PET/CT does not replace conventional imaging for initial staging of chronic disseminated candidiasis but should be performed after 3 months of antifungal therapy [109].

14. **Tuberculosis and other mycobacterioses**

The morbidity and mortality of tuberculosis (TB) remains high, despite the progress in understanding the pathogenesis and in imaging. Currently, [^18^F]FDG PET/CT is under-utilized for evaluation of TB and other mycobacterioses in the clinical setting, most likely because of cost and limited availability in countries with high prevalence of these pathologies [110].

#### Indications

- Assessment of extrapulmonary TB (EPTB) disease extent.
- Assessment of treatment response and identification of TB patients at high risk of relapse.

#### Specific protocol points and interpretation criteria

- TB can mimic malignancy, and thus [^18^F]FDG cannot be used for assessment of single pulmonary nodules. Histopathological confirmation should be obtained.

#### Diagnostic performance

- A prospective multicenter study in 358 HIV-negative patients referred for assessing EPTB demonstrated high sensitivity in detecting previously unknown sites of disease involvement, most commonly lymph nodes, bones, brain, and pleura. Furthermore, in 28% of these patients [^18^F]FDG PET/CT showed concomitant pulmonary lesions suggestive of TB [111].
- Amongst patients with latent TB, [^18^F]FDG PET/CT can identify the sub-group with subclinical disease who are at higher risk of progressing to active TB [112].
- A systematic review and meta-analysis demonstrated that [^18^F]FDG PET/CT has a potential role in the assessment of response to anti-tuberculous drug treatment and treatment outcome [113], including in patients with multidrug-resistant TB [114].
- In patients with pulmonary TB, [^18^F]FDG PET/CT correlated better with the clinical response to anti-tuberculous drug treatment than bacterial counts in sputum [115].
- The role of [^18^F]FDG PET/CT for response assessment in EPTB has been demonstrated by prospective multicentric studies [116, 117]. At treatment completion, most patients considered cured according to the current standard of care still have significant residual uptake in their lesions [117, 118].
- Uptake in TB lesions at the end of treatment could predict relapse [119, 120].

#### Areas of potential research

- To evaluate the bronchial spread of TB [121].
- To identify latent TB that can progress to active disease, with potential therapeutic implications.

#### Infection summary (Table 13)

**Table 13:** Summary of most recent published meta-analyses in the diagnosis of infectious disorders

Clinical condition	Author (year)	No studies (patients)	Sensitivity°	Specificity°	Positive likelihood ratio°	Negative likelihood ratio°	DOR°	Additional parameters°
Suspected infection in critically ill patients	Huang [16] (2020)	4 (87)	94% (79-99)	66% (45-83)	NR	NR	2.8 (1.3-4.2)	Change in management: 41% (15-66) Contribution to diagnosis: 65% (55-74)
Suspected spinal infection	Treglia [23] (2020)	26 (833)	95% (89-98)	91% (78-97)	4.7 (2.9-7.7)	0.11 (0.07-0.16)	63 (29-139)	
Non-complicated osteomyelitis	Llewellyn [27] (2019)	16 (656)	85% (72-93)	93% (83-97)	NR	NR	39 (18-83)	
Fracture-related infection	Zhang [32] (2021)	6 (NR)	89% (81-94)	78% (72-84)	4.1 (3.1-5.4)	0.14 (0.08-0.25)	29 (14-61)	AUC: 0.93 (0.90-0.95)
Diabetic foot infection	Llewelynn [41] (2020)	6 (NR)	84% (53-96)	93% (76-98)	NR	NR	34 (12-98)	
Prosthetic joint infection	Hu [46] (2022)	23 (1437)	85% (76-91)	86% (78-91)	6.1 (3.8-9.7)	0.17 (0.11-0.28)	35 (17-74)	AUC: 0.92 (NR)
PVE	Wang [67] (2020)	15 (634)	86% (81-89)	84% (79-88)	3.2 (1.7-5.9)	0.21 (0.14-0.31)	22 (10-48)	
NVE	Kamani [77] (2020)	7 (351)	36% (22-54)	99% (89-100)	8.3 (3.7-18)	0.6 (0.27-1.3)	15 (6.1-38)	
CIED (overall)	Mahmood [82] (2019)	14 (492)	85% (80-89)	90% (84-94)	NR	NR	NR	
VAD infection	Ten Hove [88] (2021)	8 (256)	95% (89-97)	91% (54-99)	3.5 (1.8-6.9)	0.14 (0.10-0.18)	38 (NR)	Driveline vs. pump/pocket results are available in this paper
Vascular graft/endograft infection	Mahmoodi [100] (2022)	10 (320)	92% (88-95)	76% (70-81)	3.5 (2.3-5.2)	0.14 (0.09-0.23)	37 (15-93)	

°: 95% confidence interval between parentheses

*DOR* diagnostic odds ratio, *NR* not reported, *PVE* prosthetic valve endocarditis, *NVE* native valve endocarditis, *CIED* cardiac implantable electronic device, *VAD* ventricular assist device, *AUC* area under the curve, *NPV* negative predictive value

### C. Inflammation

1. **Large vessel vasculitis and polymyalgia rheumatica**

Large vessel vasculitides (LVV) are a group of diseases characterized by inflammation of the medium- and large size arteries. The two main subtypes are giant cell arteritis (GCA) in patients above 50 years of age and Takayasu arteritis (TA) in younger patients, primarily below the age of 40. GCA can affect only the cranial arteries (C-GCA) presenting with the classic symptoms of headache, scalp tenderness, jaw claudication and sudden visual loss, only the aorta and its main branches (LV-GCA), or a combination of both. Polymyalgia rheumatica (PMR), an inflammatory condition affecting joints, bursae and tendons frequently co-exists with GCA. Therefore, these entities are considered as a disease continuum [122]. The classification criteria for GCA have been updated in 2022 and now include [^18^F]FDG uptake in the aorta in the point-based criteria [123]. Imaging guidelines have been published in 2018 [124] and 2023 [125].

#### Indications

- Suspected LV-GCA and TA based on key symptoms and suggestive clinical findings [125].
- Suspected C-GCA, particularly when a digital PET device is used [125–127].
- In patients with known or suspected PMR to confirm or exclude co-existing GCA [128].
- To confirm or exclude flare or recurrence of LVV based on clinical/biochemical suspicion [125].

#### Specific protocol points and interpretation criteria (refer to published guidelines [124, 125])

- Uptake time following [^18^F]FDG PET/CT injection should be a minimum of 60 minutes, and preferably 90-120 minutes [125].
- Acquisition should encompass a vertex-to-knees FOV and should be performed with arms next to the body.
- Increased acquisition time of cranial FOV and high-resolution reconstruction are recommended for assessment of cranial arteries.
- For large arteries, a 4-point visual grading scale of vascular uptake has been recommended based on a 60 minute post-injection acquisition [124].
  o Grade 0: no uptake (≤ mediastinum) and Grade 1: uptake < liver uptake, are negative for LVV.
  o Grade 2: vascular uptake = liver uptake, may be indicative of LVV. However, with digital PET, many patients without LVV have grade 2 activity in the aorta, which should therefore be interpreted with caution in the context of normal variants.
  o Grade 3: vascular uptake > liver uptake, positive for LVV.
- For cranial arteries, a 3-point visual grading scale has been used with reference to surrounding tissues, with grades 1 and 2 positive for GCA [129]:
  o Grade 0: no uptake above surrounding tissue.
  o Grade 1: uptake just above surrounding tissue.
  o Grade 2: uptake significantly above surrounding tissue.
- For PMR, refer to composite scores [124, 130, 131].

#### Diagnostic performance (Tables 14, 15, and 16)

Additional data:

- In 30 patients with suspected GCA, addition of [^18^F]FDG PET/CT increased the diagnostic accuracy from 54% to 71% and changed treatment in 27% of patients [134].
- Digital PET/CT devices show the highest reported sensitivity for detection of cranial artery GCA [135].
  **Table 14:** Major performance values of meta-analyses for diagnostic accuracy of [^18^F]FDG PET/CT in LVV

Authors	No. studies (patients)	Sensitivity (95% CI)	Specificity (95% CI)	Likelihood Ratio + (95% CI)	Likelihood Ratio - (95% CI)	DOR (95% CI)
Lee et al. [132] (2016)	3 (56)	84% (72-92)	87% (73-96)	5.2 (2.4-11.3)	0.20 (0.11-0.37)	27.2 (8.6-86.6)
Moreel et al. [126] (2023)	3 (149)	82% (61-93)	79% (60-90)	3.9 (2.1-7.3)	0.23 (0.10-0.50)	NR

**Table 15:** Major performance values of meta-analysis for diagnostic accuracy of [^18^F]FDG PET/CT for cranial artery GCA

Authors	No. studies (patients)	Sensitivity (95% CI)	Specificity (95% CI)	Likelihood Ratio + (95% CI)	Likelihood Ratio - (95% CI)	DOR (95% CI)
Moreel et al. [126] (2023)	3 (149)	58% (45-71)	97% (91-99)	18.7 (6.0-58.3)	0.43 (0.31-0.59)	NR

**Table 16:** Major performance values of meta-analyses for treatment monitoring with [^18^F]FDG PET/CT in LVV

Authors	No. studies (patients)	Sensitivity (95% CI)	Specificity (95% CI)	Likelihood Ratio + (95% CI)	Likelihood Ratio - (95% CI)	DOR (95% CI)	Comments
Van der Geest et al. [133] (2021)	4 (111)	77% (57-90)	71% (47-87)	2.7 (1.2-6.1)	0.32 (0.13-0.80)	8.3 (1.6-44.0)	Report addresses detection of relapsed/refractory disease

#### Areas of potential research

- The impact of high dose IV glucocorticoids administered for less than 3 days.
- Monitoring treatment in LVV, possibly using imaging-based composite scores.
- Prediction of disease relapse.
- PET/MRI and digital PET and/or large FOV PET performance indices for the diagnosis of C-GCA.

2. **Sarcoidosis**

Sarcoidosis is a multisystemic inflammatory disease associated with a broad clinical presentation, ranging from incidental findings in otherwise asymptomatic patients to sudden cardiac death, depending on organ involvement and disease severity [136]. Diagnosis of sarcoidosis relies on suspicious clinical presentation, exclusion of alternative causes and the presence of non-caseating granulomas on tissue samples, with additional specific criteria for cardiac sarcoidosis [136–138].

#### Indications

- Suspected clinical diagnosis in cases of equivocal prior investigations.
- Assessment of disease extent.
- Assessment of pulmonary disease activity.
- Assessment of cardiac sarcoidosis in the following scenarios:
  o Biopsy proven extra-cardiac sarcoidosis with abnormal screening for cardiac involvement [137, 139].
  o Unexplained new conduction abnormality in patients below the age of 60 [137, 139].
  o Assessment of response to therapy in cardiac sarcoidosis [139].

#### Specific protocol points and interpretation criteria

- Acquisition FOV should be expanded in specific settings such as involvement of peripheral bones.
- To allow assessment of known or suspected cardiac involvement:
  o Myocardial suppression protocol preparation is critical.
  o Resting myocardial perfusion imaging should be performed [139].
- Cardiac sarcoidosis presents as focal or focal on diffuse uptake. Diffuse, isolated homogenous basal lateral wall, papillary muscle, or basal ‘ring’ uptake most frequently represents physiological uptake related to poor suppression.

#### Diagnostic performance (Table 17)

Additional data:

- [^18^F]FDG PET/CT enables detection of unsuspected sites of extrapulmonary sarcoidosis [142].
- In prospective studies, [^18^F]FDG PET/CT identified more extensive disease compared to CT, mainly in bone and bone marrow, spleen, liver, and abdominal lymph nodes [143, 144].
- In pulmonary sarcoidosis, [^18^F]FDG PET/CT demonstrates active lesions and guides therapeutic choices [145–147].
- A prospective study in patients refractory to conventional therapy suggests that [^18^F]FDG PET/CT is useful in predicting response to advanced therapies [148].
- Sensitivity for diagnosis of cardiac sarcoidosis is improved when specific preparation protocols are used (see section "Patient preparation and precautions, no.5") [149], as well as by adding rest myocardial perfusion imaging [140].
  o A meta-analysis comparing [^18^F]FDG PET/CT and MRI for cardiac sarcoidosis reports higher sensitivity for MRI and comparable specificity. When excluding patients receiving anti-inflammatory therapy, the sensitivity of [^18^F]FDG PET/CT was significantly higher and comparable to that of MRI [141], likely because [^18^F]FDG uptake is an indicator of active inflammation.
- In cardiac sarcoidosis, [^18^F]FDG PET/CT enables risk stratification. Abnormal uptake was associated with increased rates of major cardiovascular adverse events (MACE), mainly in cases with right ventricular involvement [150].
  **Table 17:** Major performance values of meta-analyses for [^18^F]FDG PET/CT in cardiac sarcoidosis

Authors	No. studies (patients)	Sensitivity (95% CI)	Specificity (95% CI)	Likelihood Ratio + (95% CI)	Likelihood Ratio - (95% CI)	DOR (95% CI)
Kim et al. [140] (2020)	17 (891)	84% (71-91)	83% (74-89)	4.9 (3.3-7.3)	0.20 (0.11-0.35)	25 (12-51)
Aitken et al. [141] (2022)	26 (1363)	84% (74-90)	82% (75-88)	NR	NR	NR

#### Areas of potential research

- Evaluation of response to second- or third-line therapies.

3. **Inflammatory bowel diseases (IBD)**

Inflammatory bowel diseases (IBD), a group of chronic relapsing disorders of the gastrointestinal (GI) tract, include Crohn’s disease (CD) and ulcerative colitis (UC) which differ in bowel location and pattern. Diagnosis is based on clinical, endoscopic and histological criteria. Treatment depends on disease severity [151, 152]. Imaging tests are performed in unclear cases to evaluate the extent and severity of disease, to diagnose early relapse or complications, and during follow-up [151, 153].

[^18^F]FDG PET/CT can evaluate disease extent at diagnosis and differentiate between fibrotic and inflammatory strictures during follow-up. Several guidelines and society recommendations for assessment of IBD provide indications [154] and interpretation criteria for [^18^F]FDG PET/CT [154, 155].

#### Indications

- Extent of IBD at diagnosis.
- Early assessment of therapy.
- Differential diagnosis between fibrotic and inflammatory stricture.

#### Specific protocol points and interpretation criteria

- Specific bowel preparation recommendations have been published in general imaging guidelines [156].
- The acquisition FOV should be limited to the abdomen and pelvis.
- Visual analysis can be hampered by imperfect registration due to bowel motion and incomplete patient preparation.
- SUV_max_ is a semi-quantitative parameter used for the evaluation of [^18^F]FDG uptake in IBD, although no defined cut-off has been identified to differentiate between positive and negative findings [153, 157, 158].

#### Diagnostic performance (Table 18)

4.  **Retroperitoneal fibrosis and IgG4-related disease**

Retroperitoneal fibrosis (RPF) is a rare collagen vascular disease with unknown etiology. It is characterized by a fibro-inflammatory reaction, usually originating around the retroperitoneal vessels and extending to the neighboring structures. Over two thirds of RPF cases are idiopathic while the rest occurs secondary to other causes. The main role of [^18^F]FDG PET/CT is to evaluate the presence of disease activity and its extent with impact on prognosis, treatment options, outcomes and treatment response assessment [159, 160].

Immunoglobulin G4 (IgG4)-related disease (IgG4-RD) is an immune-mediated condition that can occur at any anatomical site, mainly affecting the salivary glands, pancreas, thyroid, lymph nodes, large vessels and lungs. [^18^F]FDG PET/CT is useful for evaluation of disease extent and potentially for treatment response assessment [161].

**Table 18:** Major performance values of meta-analyses for [^18^F]FDG PET/CT in inflammatory bowel disease

Authors	No. Studies (patients)	Sensitivity (95% CI)	Specificity (95% CI)	Likelihood Ratio + (95% CI)	Likelihood Ratio - (95% CI)	DOR (95% CI)
Treglia et al. [153] (2013)	19 (454)	85% (81-88)	87% (84-90)	6.2 (2.9-13.4)	0.19 (0.10-0.34)	44.4 (11.8-167.1)

#### Indications

- Assessment of RPF disease activity, particularly in asymptomatic patients with acute phase reactant increase [159, 160].
- Assessment of disease extent in IgG4-RD [161].

#### Indications with insufficient evidence

- Diagnosis of IgG4-RD.

#### Specific protocol points and interpretation criteria

- RPF: Abnormal uptake in retroperitoneal tissue involving the abdominal aorta and adjacent structures.
- IgG4-RD: Abnormal uptake in one of the common sites of disease mentioned above.

#### Diagnostic performance

Systemic descriptive reviews are available for RPF [160] and IgG4-RD [161], but no meta-analyses are available Table 19.

#### Inflammation summary (19)

**Table 19:** Summary of most recent published meta-analyses in the diagnosis of inflammatory disorders

Clinical condition	Author (year)	No studies (patients)	Sensitivity°	Specificity°	Positive likelihood ratio°	Negative likelihood ratio°	DOR°	Additional parameters°
LVV	Moreel [126] (2023)	3 (149)	82% (61-93)	79% (60-90)	3.9 (2.1-7.3)	0.23 (0.10-0.50)	NR	
Cranial artery GCA	Moreel [126] (2023)	3 (149)	58% (45-71)	97% (91-99)	19 (6.0-58.3)	0.43 (0.31-0.59)	NR	
Cardiac sarcoidosis	Aitken [141] (2022)	26 (1363)	84% (74-90)	82% (75-88)	NR	NR	NR	
Inflammatory bowel disease	Treglia [153] (2013)	19 (454)	85% (81-88)	87% (84-90)	6.2 (2.9-13.4)	0.19 (0.10-0.34)	44 (12-167)	AUC: 0.93 (0.87-1.00)

°: 95% confidence interval between parentheses

*DOR *diagnostic odds ratio, *NR *not reported, *LVV *large vessel vasculitis, *GCA *giant cell arteritis, *AUC *area under the curve

### D. Other [^18^F]FDG infection and inflammation indications with insufficient evidence to date

- COVID-19 including long-COVID [162]
- Interstitial lung diseases [163]
- Inflammatory arthropathies (e.g. rheumatoid arthritis, psoriatic arthritis, ankylosing spondylitis, spondyloarthropathies) and myopathies [164]

## Qualifications and responsibilities of personnel

In the United States, see the SNMMI guideline for general imaging [165]. In Europe, the certified nuclear medicine physician who performs the study and signs the report is responsible for the procedure, according to national laws and rules.

## Procedure/specifications of the examination

In general, [^18^F]FDG PET/CT studies in patients with known or suspected infection and inflammation follow the recommendations for imaging malignancies, as well as general patient preparation instructions and common pitfalls detailed in the most recently published 2015 EANM guidelines for [^18^F]FDG PET/CT for tumor imaging version 2.0 [1].

### Request

The request for the examination should include all relevant medical information, justifying the clinical need to perform an [^18^F]FDG PET/CT study, including a known or suspected diagnosis, relevant patient history and the specific question of the referring physician.

Results of relevant laboratory tests and prior imaging studies including radiographs, ultrasound, CT, MRI, and [^18^F]FDG PET/CT or their written reports, should be available for comparison. Knowledge of prior treatment including surgery, radiation therapy, immunotherapy, antibiotic and glucocorticoid therapy and their timing are essential.

### Patient preparation and precautions

Present document underscores specific issues related to [^18^F]FDG imaging of infection and inflammation. Some of the known general preparatory measures may not be needed when limited FOV studies are performed, in particular to evaluate localized infectious processes in the lower extremities.

1. Pregnancy (suspected or confirmed)

In the case of a patient who is known or suspected to be pregnant, the decision for performing the test has to be agreed in consensus by the patient, referring physician and the imaging expert. In non-urgent cases, a pregnancy test may help with the decision to postpone the study, provided the 10-day post-ovulation blackout is understood and adopted.

2.  Breastfeeding

Interruption of breastfeeding after [^18^F]FDG administration is not required since little is excreted in the milk [166]. Contact between the mother and child should be avoided or at least restricted for 4 hours after injection of [^18^F]FDG to reduce the radiation dose the infant receives from exposure to the mother [167].

3.  Diabetes and serum glucose level before [^18^F]FDG administration

[^18^F]FDG and glucose compete for the same transporters. High serum glucose levels can therefore potentially interfere with radiotracer uptake in target sites and it has been therefore recommended that [^18^F]FDG should be administered when blood glucose levels are below 11 mmol/L [1]. While in a group of patients with suspected infection neither diabetes nor hyperglycemia had any significant impact on the false negative rate of [^18^F]FDG imaging [168], more recently, an inverse relation has been shown between the yield of [^18^F]FDG-PET/CT and glycemia in patients with bacteremia [169]. In patients with severe, poorly controlled diabetes, a population often associated with infection, all efforts should be made to decrease blood glucose values to the lowest possible level, e.g. by appropriate study scheduling for late morning, approximately 4 hours after breakfast. Recording blood glucose levels at the time of injection is mandatory prior to radiotracer administration. The time interval between various types of insulin and [^18^F]FDG administration should follow published recommendations [1]. Metformin increases intestinal glucose uptake and colonic [^18^F]FDG activity [170] which can mask adjacent sites of abdominal infection or inflammation. Holding metformin for 48 hours may improve assessment of the bowel and abdomen [171], but withholding is not necessary when the abdomen is not in the imaged FOV or when is not the main clinical region of interest.

4. Kidney and liver failure

[^18^F]FDG is primarily eliminated through the kidneys. This hampers its utility for detecting urinary tract infections. Image quality may be suboptimal in patients with kidney failure [172–174]. If contrast-enhanced CT is planned to be part of the PET/CT study, all precautions necessary for IV iodine contrast material administration should be followed [175].

Diffuse increased hepatic [^18^F]FDG activity has been described in patients with liver failure, with no clear evidence whether these findings affect the diagnostic accuracy in hepatic infectious or inflammatory processes [173]. However, caution is needed when using a visual scoring system in which liver uptake serves as reference activity.

5. Myocardial suppression protocol

As a glucose analogue, [^18^F]FDG accumulates in the normal myocardium. Thus, specific protocols are required to minimize the physiologic [^18^F]FDG uptake in the heart in cases of a known or suspected infectious or inflammatory process located in the myocardium or nearby anatomical structures or cardiac devices. Optimal myocardial suppression improves the diagnostic accuracy of [^18^F]FDG PET/CT for diagnosing cardiac sarcoidosis [149] and IE [77]. A multitude of myocardial suppression protocols have been proposed [176]. Current recommendations suggest a prolonged fasting period of at least 12 hours preceded by a high fat-low/no carbohydrate-diet for 24-48 hours, with or without the administration of intravenous heparin (50 IU/kg) 15 min before tracer injection [62, 177, 178]. Despite the use of these preparation protocols, suboptimal myocardial suppression may be observed in 5-20% of patients [176]. Emerging evidence suggests that beta-hydroxybutyrate serum levels could be used to indicate if a patient has reached adequate ketosis following a myocardial suppression preparation [179].

6. Glucocorticoids

Glucocorticoids are a mainstay for treating inflammatory conditions that are currently often evaluated with [^18^F]FDG-PET/CT at both diagnosis and during follow-up. In general, it is recommended to perform the study prior to starting treatment (unless there is a risk of complications) since glucocorticoid administration can rapidly reduce [^18^F]FDG uptake. False negative results following steroid treatment have been described mainly in GCA and other systemic vasculitides [180]. However, more recent studies have shown that high dose oral glucocorticoids do not significantly affect the diagnostic accuracy within the first few days after treatment onset in patients with large vessel arteritis, rheumatoid arthritis, and PMR. As such, an open “diagnostic window” of up to three days after the beginning of treatment has been proposed. There was no decrease in sensitivity when comparing studies in untreated patients with those receiving glucocorticoids for 3 days or less despite a decrease in uptake intensity of up to 15% [181, 182]. After approximately 10 days, a more significant reduction in the intensity of uptake, up to 40% was reported [182] resulting in a correct diagnosis in only one-third of cases [182, 183]. The use of IV glucocorticoids with more rapid reduction in inflammation may shorten this “diagnostic window” to less than 3 days. Very few studies have been specifically designed to investigate if and how long glucocorticoids should be discontinued prior to [^18^F]FDG-PET/CT. One study in PMR patients showed that a controlled reduction of prednisolone dose for 1 week followed by a 1 week discontinuation (in patients that had been treated for 8 weeks) significantly enhanced the sensitivity from 36% to 66%, but remained lower compared to baseline [^18^F]FDG-PET/CT prior to treatment. Based on limited data and expert opinion, a discontinuation of glucocorticoids for at least 2 weeks can be considered if clinical presentation allows. The safety and utility for other indications has not been validated [184].

7. Antibiotics

Studies should preferentially be performed prior to the beginning of antibiotic treatment or as soon as possible thereafter, however, without delaying treatment initiation. [^18^F]FDG PET/CT studies performed during antibiotic treatment in patients with suspected infection should be interpreted with caution. A decrease in the intensity and a change in the distribution pattern of [^18^F]FDG uptake in known infectious processes are parameters used to assess the results of antibiotic therapy. [^18^F]FDG PET/CT has been reported to correctly identify foci of increased uptake compatible with infection in all studies performed in patients with microbiologically documented infections receiving appropriate antibiotic therapy, with no false negative results. Positive study results were reported in a small number of patients with severe disease showing a lack of response even after receiving appropriate antibiotic therapy for a duration of one month [185].

8. Evaluation of critically ill patients

The management of such patients is a logistical and technical time-consuming challenge for the nuclear medicine department staff. It requires the on-site presence of a highly trained multidisciplinary team. When scheduling a test in such a patient, logistics, nursing and medical care should be prepared well in advance with regards to general but also specific recommendations, including radiotracer administration within the ICU unit and use of large FOV PET/CT systems when available can minimize the time spent away from the ICU unit [186].

9. Specific instructions for [^18^F]FDG PET/CT imaging in inflammatory or infectious diseases (in addition to the general recommendations described in the guidelines for [^18^F]FDG PET/CT [1]).

- When a process involving the heart is known or suspected, a dedicated myocardial suppression regimen is recommended to suppress physiological myocardial uptake and adherence to cardiac regimen prior to FDG administration should be verified (see "Patient preparation and precautions, no.5").
- Record the administration and duration (start of treatment) of specific drugs that may interfere with [^18^F]FDG uptake such as antibiotics or glucocorticoids.
- The following parameters must be checked, known and recorded:
  - Fever and/or elevation of acute inflammatory markers such as ESR or CRP.
  - Diabetes and its ongoing treatment.
  - History of trauma, recent surgery or invasive diagnostic procedures performed within the last 4 weeks.
  - Neoplastic disorder, recent chemo- and radiotherapy that may influence the interpretation of a procedure performed in the framework of infection and inflammation.
  - Known immunosuppressive status.
  - Recent vaccination and site of injection.
  - Presence of a known infectious or inflammatory condition.

### Radiopharmaceutical administration

1. The radiopharmaceutical should be administered through an IV line. The administered activity varies according to local regulations, in addition to patient characteristics, indication for study, type of imaging device and acquisition protocol.
2. Uptake period after injection: a minimum 60-minute interval between [^18^F]FDG injection and acquisition is recommended to achieve an adequate radiotracer biodistribution. A preferred uptake period of 90 minutes and/or the addition of delayed images can be applied for vasculitis, cardiac sarcoidosis or IE.
3. Post-procedure recommendations:

At the end of the imaging procedure the technical quality of the study must be checked by the technologist and approved by the nuclear medicine physician. Patients can then resume their normal routine without further precautions.

### Radiation exposure

The effective dose for [^18^F]FDG is 1.9x10^-2^ mSv/MBq [166]. In addition, radiation exposure from the CT, which depends on the type of study, diagnostic CT vs low-dose, needs to be considered.

### Image acquisition protocol

Specific acquisition protocols are discussed in appropriate sections as needed.

- Cardiac: delayed dedicated static (>90 minutes post-injection) and cardiac gated acquisition can improve the diagnostic accuracy for selected indications [187].
- In patients with suspected or known involvement of the lower extremities the acquisition FOV should include the feet. The upper extremities should be included in selected cases when clinically relevant.
- For specific clinical indications such as assessment of a suspected PJI or a diabetic foot infection, imaging can be confined to one or two FOVs.
- Recent technological advances in PET equipment have enabled a further reduction of injected activity and/or acquisition duration in cases with infection and inflammation, similar to cancer patients, while maintaining excellent image quality [188, 189], resulting in a subsequent decrease in radiation exposure. Total-body PET/CT systems can be potentially used for single-step whole-body PET images in an expanding, wider range of patient populations, including critically ill and debilitated patients with suspected or known infectious processes.
- CT acquisition parameters are detailed in the EANM tumor imaging guidelines [1].
- Metallic artifact reduction techniques should be used whenever available and indicated.
- For peripheral musculoskeletal infection indications, CT should be performed for a limited FOV, with thin slice acquisition and reconstruction with bone matrix in all orthogonal planes.

### Image analysis and interpretation

1. Physiologic ^18^F-FDG distribution, relevant for evaluation of infection and inflammation:

- In the fasting state without any specific myocardial suppression protocol, variable [^18^F]FDG uptake can be observed in the myocardium.
- [^18^F]FDG is excreted via the kidneys and accumulates in the urinary tract.
- Variable uptake can be found in skeletal muscles, depending on recent physical activity and insulin administration.
- Uptake in the GI tract is highly variable and can be influenced by ongoing treatment with metformin or analogs.
- Uptake in the lymphoid tissue and normal size lymph nodes can be variable and is non-specific [1, 190].
- Diffuse bone marrow and splenic uptake can be noted in the presence of active infection or inflammation, and other conditions [191].

2. Qualitative, visual analysis

- PET images are evaluated for abnormal sites of increased uptake according to their intensity and uptake patterns (focal, linear, diffuse, heterogeneous). In general, a positive study shows increased uptake in a lesion, with an intensity higher than the surrounding background and not explained by physiological activity. A grading score has been described for various indications to standardize interpretation. Findings are then correlated with location and morphologic data obtained from the CT component. Specific criteria are described in the “interpretation criteria” section for various clinical indications.
- Radiotracer avidity in loco-regional lymph nodes has been suggested as a predictor of an infectious process but its role as a specific interpretation criterion is not known and should therefore be used with caution [192, 193].
- The CT component should be reviewed for findings supporting the suspected diagnosis or other causes of [^18^F]FDG uptake.
- In musculoskeletal infections:
  - Accurate co-registration of PET and CT images is of utmost importance to evaluate for the presence of osteomyelitis.
  - In cases with intense soft tissue uptake and suspected blooming into adjacent bone, the intensity of the window to define the epicenter of the lesion should be adjusted to evaluate for bone involvement.
  - CT findings should be assessed for signs of acute and chronic osteomyelitis [194].
  - Signs of fracture, arthropathy, metastases and degenerative changes, if present, can indicate a differential diagnosis for increased [^18^F]FDG uptake.

- In view of potential false negative [^18^F]FDG PET results, it is essential that even in cases of a negative PET study, the CT component should be thoroughly evaluated. In general, causes for false-negative [^18^F]FDG PET results are related to:
  - Size of the lesion.
  - Location of lesions adjacent to sites of high physiologic activity.
  - Intake of drugs interfering with uptake.

Low quality studies should be specifically recorded. Interpretation can be potentially impaired, in particular in scenarios such as:

- Obese patients.
- Altered biodistribution.
- Patient movement between the PET and CT acquisitions, resulting in incorrect fusion.
- Patients with metallic hardware and no or inappropriate software correction.
- In the presence of metallic hardware, when older PET/CT devices are used and in case of doubt, both non-attenuated (NAC) and attenuation corrected (AC) images should be reviewed to identify metal induced artifacts on AC images. Such artifacts are infrequent with more recent PET/CT devices.

3. Quantitative analysis (SUV)

Unlike for its use in oncology, SUV or target-to-background (T/B) ratios have not been generally validated in the field of infection to allow differentiation from a sterile inflammation or malignancy [60] and should therefore be used with caution both at diagnosis and during treatment evaluation.

## Areas of future research and perspectives

Research should preferably be designed in the framework of multicenter studies with standardized data collection and interpretation, consecutive recruitment of patients and proper blinding of test assessors for appropriate health technology assessment. Where appropriate, patient compliance and patient outcomes should be also evaluated.

- Assess the potential role for late imaging (90-180 minutes post [^18^F]FDG injection) in selected indications such as osteomyelitis, vascular and cardiac imaging aiming at improving image quality through higher target to background ratios.
- Assess the role of ECG-synchronized cardiac gated acquisition in suspected endocarditis.
- Assess the added value and potential improvement of diagnostic yield using IV contrast with [^18^F]FDG PET/CT in selected indications.
- Define the role and threshold(s) for SUV or lesion-to-background ratios to diagnose and differentiate an infection from a sterile inflammation or a malignant process.
- Determine whether the diagnostic accuracy is further improved with new digital or large FOV hybrid PET systems, particularly in the evaluation of small lesions, while also reducing the administered radiotracer activity.
- Determine the potential added value of PET/MRI for assessment of infectious processes in general and specifically for indications such as spondylodiscitis, diabetic foot infection, osteomyelitis, polycystic disease, cardiac sarcoidosis, cranial artery vasculitis and IBD.
- Compare the diagnostic accuracy of [^18^F]FDG imaging vs other modalities such as WBC SPECT/CT and MRI in various indications. This should include defining the appropriate choice between these tests considering their performance indices as well as local availability, expertise and cost effectiveness.
- Understand the impact of antibiotic therapy and its duration prior to imaging on the diagnostic accuracy.
- Monitoring therapy response remains one of the most important but insufficiently studied potential additional applications of [^18^F]FDG PET imaging in infection and inflammation. Appropriate interpretation criteria and added value needs to be validated for several indications.
- Identify the optimal time point for integrating [^18^F]FDG PET/CT in the diagnostic work-up of infectious and inflammatory processes in terms of cost-effectiveness.
- Evaluate the role of artificial intelligence for [^18^F]FDG PET/CT in the assessment of infectious and inflammatory diseases.

## Supplementary Information

Below is the link to the electronic supplementary material.

Supplementary file1 (DOCX 15 KB)

## Data Availability

Data are available upon request.

## Liability

This guideline summarizes the views of the EANM inflammation and infection committee and the SNMMI. It reflects recommendations for which the EANM and SNMMI cannot be held responsible. The recommendations should be taken into context of good practice of nuclear medicine and do not substitute for national and international legal or regulatory provisions.
