# Supplementary material for: EANM/SNMMI guideline/procedure standard for [18F]FDG hybrid PET use in infection and inflammation in adults v2.0
Source: Eur J Nucl Med Mol Imaging. 2024 Oct 10;52(2):510–38. doi: 10.1007/s00259-024-06915-3 (PMC11732780; doi:10.1007/s00259-024-06915-3)
Supplement: Supplementary file 1 — Supplementary file1 (DOCX 15 KB) [file 259_2024_6915_MOESM1_ESM.docx]

**PREAMBLE**

The European Association of Nuclear Medicine (EANM) is a professional non-profit medical association that facilitates communication worldwide between individuals pursuing clinical and research excellence in nuclear medicine. The EANM was founded in 1985. The Society of Nuclear Medicine and Molecular Imaging (SNMMI) is an international scientific and professional organization founded in 1954 to promote the science, technology, and practical application of nuclear medicine. Its 15,000 members are physicians, technologists, and scientists specializing in the research and practice of nuclear medicine. In addition to publishing journals, newsletters, and books, the SNMMI also sponsors international meetings and workshops designed to increase the competencies of nuclear medicine practitioners and to promote new advances in the science of nuclear medicine.

The EANM/SNMMI will periodically define new standards/guidelines for nuclear medicine practice to help advance the science of nuclear medicine and to improve the quality of service to patients. Existing standards/guidelines will be reviewed for revision or renewal, as appropriate, on their fifth anniversary or sooner, if indicated. Starting February 2014, the SNMMI guidelines have been referred to as procedure standards. Any practice guideline or procedure guideline published before that date is now considered an SNMMI procedure standard.

Each standard/guideline, representing a policy statement by the EANM/SNMMI, has undergone a thorough consensus process in which it has been subjected to extensive review. The EANM/SNMMI recognizes that the safe and effective use of diagnostic nuclear medicine imaging requires specific training, skills, and techniques, as described in each document.

The EANM and SNMMI have written and approved these standards/guidelines to promote the use of nuclear medicine procedures with high quality. These standards/guidelines are intended to assist practitioners in providing appropriate nuclear medicine care for patients. They are not inflexible rules or requirements of practice and are not intended, nor should they be used, to establish a legal standard of care. For these reasons and those set forth below, the EANM/SNMMI cautions against the use of these standards/guidelines in litigation in which the clinical decisions of a practitioner are called into question.

Medical professionals taking into account the unique circumstances of each case must make the ultimate judgment regarding the propriety of any specific procedure or course of action. Thus, there is no implication that an approach differing from the standards/guidelines, standing alone, is below the standard of care. To the contrary, a conscientious practitioner may responsibly adopt a course of action different from that set forth in the standards/guidelines when, in the reasonable judgment of the practitioner, such course of action is indicated by the condition of the patient, limitations of available resources, or advances in knowledge or technology subsequent to publication of the standards/guidelines.

The practice of medicine involves not only the science but also the art of dealing with the prevention, diagnosis, alleviation, and treatment of disease. The variety and complexity of human conditions make it impossible to always reach the most appropriate diagnosis or to predict with certainty a particular response to treatment. Therefore, it should be recognized that adherence to these standards/guidelines will not ensure an accurate diagnosis or a successful outcome. All that should be expected is that the practitioner will follow a reasonable course of action based on current knowledge, available resources, and the needs of the patient to deliver effective and safe medical care. The sole purpose of these standards/guidelines is to assist practitioners in achieving this objective.
